# Product Development Studies on Sonocrystallized Curcumin for the Treatment of Gastric Cancer

**DOI:** 10.3390/pharmaceutics7020043

**Published:** 2015-04-27

**Authors:** Mohammad Ashif Khan, Nida Akhtar, Vijay Sharma, Kamla Pathak

**Affiliations:** Department of Pharmaceutics, Rajiv Academy for Pharmacy, N.H. #2, Delhi Mathura Road, P.O. Chhattikara, Mathura-281001, India; E-Mails: mohammadashif212@gmail.com (M.A.K.); nidakhtr378@gmail.com (N.A.); vijay_ceutics07@yahoo.co.in (V.S.)

**Keywords:** curcumin, melt sonocrystallization, spectral characterization, *in vitro* cytotoxicity, gastroretentive floating tablet

## Abstract

Curcumin suffers from the limitation of poor solubility and low dissolution that can lead to limited applications. The investigation was aimed to substantiate the potentiality of melt sonocrystallized gastroretentive tablets of curcumin. Melt sonocrystallized curcumin (MSC CMN) was developed and its therapeutic potential was validated by *in vitro* cytotoxicity studies against Human oral cancer cell line KB. MSC curcumin was then formulated as floating tablet and evaluated. MSC form of CMN exhibited 2.36-fold and 2.40-fold solubility enhancement in distilled water and phosphate buffer, pH 4.5, respectively, better flow properties and intrinsic dissolution rate (0.242 ± 1.42 and 0.195 ± 1.26 mg/cm^2^/min) in comparison to its original form. The GI_50_ value of MSC CMN was found to be less than 10, specifying inhibition of growth more effectively at its least concentration by 50%. The gastroretentive-floating tablet (Formulation F4) displayed controlled drug release (96.22% ± 1.43%) for over 12 h. The present study revealed melt sonocrystallization can be used to produce particles with superior biopharmaceutical properties without the use of organic solvents or the addition of other excipients, and amenable to formulation in to a pharmaceutical dosage form.

## 1. Introduction

Curcumin is an anti-inflammatory, antimicrobial and anticancer drug, specifically used in the treatment of gastric cancer [1]. Despite the fact that curcumin is therapeutically acclaimed it suffers from the limitations of poor solubility and dissolution that can lead to limited applications [2]. The extremely low solubility of curcumin (0.6 µg/mL) [3] results in low bioavailability and hence poor clinical efficacy [4]. Its low solubility in water as well as in acidic environment results in poor dissolution characteristics, hence decline in its availability [5]. Various approaches have been used to enhance the solubility of curcumin. These include solid dispersion technique [6]; floating microspheres [7] and nanoformulations [8] like nanoemulsions, nanospheres and nanoparticles [9]. Solid dispersion methodology is reported to be a time-consuming approach due to long processing and drying time. The use of organic solvents in this technique may produce toxicity in final product [10]. On the other hand, floating microspheres have limited drug loading potential due to higher need of excipients [11]. Nanoemulsions present stability problems as their stability is temperature and pH dependent [12]. In case of nanospheres, physical handling is difficult in liquid as well as in dry forms. Chance of particle aggregation may increase due to smaller size and larger surface area of nanospheres. Also, these carriers have limited drug loading [13]. Production of nanoparticles involves the use of polyvinyl alcohol as a detergent that may present toxicity issues, possess limited targeting abilities and cytotoxicity has also been reported in some cases [14]. Recently various developments have been made to improve the solubility as well as dissolution characteristics of curcumin. Co-crystals of curcumin have also been prepared using liquid-assisted grinding, and evaluated for dissolution rates. Co-crystals curcumin−pyrogallol and curcumin−resorcinol dissolution rate was 12 and 5 times faster compared to the commercially available curcumin [15]. Mesoporous silica nanoparticles encapsulating curcumin have been developed by Jambhrunkar *et al.* (2014) demonstrated improved solubility, *in vitro* release profile and remarkably improved cell cytotoxicity compared to the pure curcumin [16]. The mesoporous silica nanoparticles (MCM-41) with characteristic surface chemistry were studied as a novel carrier system to depict their influence on drug delivery and anticancer activity of curcumin. It was observed by the authors that both positively as well as negatively charged surface showed improved drug release and anticancer activity when compared to pure curcumin. Higher cellular uptake was observed in comparison to negatively charged nanoparticles due to electrostatic interaction with cells. The limitation observed with the hydrophobic surface modified nanoparticles (MCM-41-CH_3_) was no improvement in drug release and anticancer activity owing to its poor wetting effect [17]. In another report, rod-like mesoporous silica nanoparticles were formulated by Xu *et al.* that showed 37% more cellular uptake and drug delivery efficacy as compared to their counterparts with a smooth surface [18]. Silica vesicles of curcumin were synthesized by Yang *et al.* using mixed triblock copolymer surfactants as the structure-directing agents, tetraethyl orthosilicate and tetrapropyl orthosilicate as mixed silica sources. Silicon phthalocyanine dichloride (SiPC) incorporated into the vesicles, showed improved delivery of SiPC into cancer cells and photodynamic therapy efficiency [19]. CUR-γ-hydroxypropyl cyclodextrin (CUR-CD) hollow spheres were formulated by Popat *et al.* and loaded into positively charged biodegradable chitosan (CUR-CD-CS) nanoparticles. These nanoparticles showed higher *in vitro* drug release and higher cytotoxicity in SCC25 cell line amongst all tested formulations. The cytotoxicity results depicted 100% apoptotic cell death in the case of nanoparticles [20].

Considering the innovations explored in this particular area, particle engineering (solvent free approach) is also utilized as a technique that modifies the physicochemical, micromeritic and biopharmaceutical properties of the drug [21]. Use of ultrasonic waves has been introduced in the last few years to increase solubility of sparingly soluble drugs. This technique has proven to be an efficient tool to influence the external appearance and structure of crystalline product [22]. It is a simple technique that can be run under ambient conditions of temperature and pressure. The cost-effective reaction vessel used for this purpose is of simple geometry that makes the cleaning process simpler for pharmaceutical requirements [23]. The nanosized and microsized crystals developed from ultrasound-assisted crystallization (sonocrystallization) yielded a powder having elastic moduli and bulk cohesions remarkably higher than macrosized crystals, thus leading to improved tabletability without the use of excipients, particle coating, salt, or co-crystal formation [24].

Thus, the project was undertaken with two objectives (i) preparation of melt sonocrystallized form of curcumin and its select preformulation studies focused on solubility analysis and bulk characterization; and (ii) product development of melt sonocrystallized form as gastroretentive system considering the sensitivity of curcumin to alkaline environment of small intestine. Thus, the aim was to develop floating tablet of melt sonocrystallized curcumin for overcoming the solubility limited dissolution at the target site for localized delivery of gastric cancer. *In vitro* cytotoxicity studies were conducted to prove efficacy of the engineered curcumin.

## 2. Materials and Methodologies

### 2.1. Materials

Curcumin (CMN) was a kind gift sample obtained from Shiv Shakti Herbals, Sikandrabad, India. HPMC K15M, potassium di-hydrogen phosphate, micro-crystalline cellulose (PH 101) and ethanol were procured from SD Fine chemicals Ltd., Mumbai, India. Liquid paraffin was purchased from Ranbaxy laboratory Ltd., Punjab, India. Citric acid and sodium bicarbonate were procured from Ranbaxy fine chemicals Ltd., New Delhi, India.

### 2.2. Methodologies

#### 2.2.1. Preparation of Melt Sonocrystallized Form

The original form of curcumin (CMN) 10 g was melted in a test tube on a paraffin oil bath maintained at 200 °C. The molten mass was poured into a beaker containing 100 mL of double distilled water maintained at 75–80 °C and sonicated for 4 min using an ultrasonic bath (Bath sonicator, 1.5 L, HICON, Grover Enterprises, New Delhi, India) at a frequency of 33 ± 3 kHz at 80% amplitude with the power rating of 60 watt. The product was separated by filtration using Whatman filter paper #1 (particle retention, 11 µm). The product was dried at room temperature overnight and kept in a desiccator to get melt sonocrystallized form of curcumin (MSC CMN).

#### 2.2.2. Evaluation

##### Particle Size and Its Distribution

The particle size of CMN and MSC CMN was determined by Dynamic laser scattering using particle size analyzer (Beckman Coulter LS 13 320, Brea, CA, USA) equipped with an argon laser. The suspension was added to the sample cell attached inside the coulter counter then started the analysis. The addition of sample drop wise in the sample cell was continued up to the obscuration rate of 10%. Particle size analysis was based on the refractive index (RI) of both the material. The size distribution was expressed as volume median diameter (VMD) and span. The data was obtained for quantification of powder in terms of skewness, kurtosis, interquartile coefficient of skewness (IQCS), specific surface area, span and variance by using the following equation [25].

IQCS = [(c − a) − (a − b)]/[(c − a) + (a − b)] (1)

where, *a* is the median diameter, *b* and *c* are the lower and upper quartile points.

Span = D (90%) − D (10%)/D (50%) (2)

where, *D* (90%) is the median diameter at 90% cumulative size, *D* (50%) and *D* (10%) cumulative size [26].

##### Flow Properties

CMN and MSC CMN forms were characterized for bulk density, tapped density, Carr’s compressibility index, angle of repose and Hausner’s ratio. Dynamic angle of repose of CMN and MSC CMN was determined by placing 1 g of drug powder in a lab fabricated rotating cylinder apparatus and allowed to rotate at 25 rpm for 5 min. The angle made by the bulk of the drug powder against the horizontal tangent was recorded and dynamic angle of repose each sample was calculated [27]. The bulk density was obtained by dividing the weight of sample by the final volume in cm^3^ of the sample contained in the cylinder [25]. Hausner’s ratio was determined by dividing the tapped density by bulk density. The percent compressibility index was calculated by using the equation:

% Compressibility Index = (Tapped density −Poured density)/Tapped density × 100 (3)

##### Equilibrium Solubility

The equilibrium study was determined by shake flask method. The solubility was determined in double distilled water and phosphate buffer, pH 4.5. Excess amount of the drug was placed in the 25 mL of conical flask. The mixture was shaken for 72 h in a thermostatic water bath shaker (HICON, Grover Enterprises, New Delhi, India) at temperature 25 ± 2 °C. The sample was filtered with the help of Whatmann filter paper #1. The filtrate was centrifuged (Remi Centrifuge, Remi Instrument Ltd., Vasai, India) for 15 min at 3000 rpm (503 g). The amount of drug dissolved was then analyzed spectrophotometrically.

##### Intrinsic Dissolution Rate

Intrinsic dissolution rate was determined by using rotating disc method [28]. Disc(s) of CMN and MSC CMN was prepared by compressing 500 mg of each with a compression force of 9-ton using 13 mm flat faced IR disc punch and compaction pressure of 600 mm Hg with dwelling time of 5 min. The top and sides of compressed disc was coated using low melting point paraffin wax (58–60 °C) and fixed to the holder of the rotating basket leaving one face to exposed (surface area = 1.131 cm^2^) that was cleared of residual wax. The dissolution of compressed disc of CMN and MSC CMN was conducted in double distilled water and phosphate buffer, pH 4.5, stirred at 100 rpm and maintained at 37 ± 0.5 °C. Five milliliters of aliquots was withdrawn and replaced with the fresh media. The aliquots were filtered by using whatmann filter paper #1, diluted and analyzed spectrophotometrically. The linear portion of each dissolution profile was used to derive the intrinsic dissolution rate [27].

(4) $$\mathrm{IDR}=\frac{a}{t}\times \frac{1}{A}$$

where, *a* is amount of dissolved drug in media (mg), *A* is surface area (cm^2^), and *t* is time (min).

##### X-ray Diffraction

Samples of CMN and MSC CMN were subjected to XRD analysis. X-ray diffraction (XRD) patterns were recorded using Advance X-ray diffractometer (Bruker D8, Karlsruhe, Germany). The samples were irradiated with mono chromatized Cu *K*α radiation, generated at 1.54239 Å wavelength, at 30 kV and 30 mA. Afterwards, the samples were step scanned between 10° and 80° at 2θ scale.

##### Scanning Electron Microscopy

The photomicrographs of both CMN and MSC CMN were obtained using scanning electron microscope (JEOL 5400, Jeol, Tokyo, Japan). Particles were coated with thin gold layer by sputter coater unit (VG Microtech, West Sussex, UK) under an argon atmosphere in order to make them conductive. The coating time was 5–6 min. Surface morphology of both the powders was studied by observing the obtained photomicrographs at an acceleration voltage of 4 kV.

##### Differential Scanning Calorimetry (DSC)

Thermal behavior of CMN and MSC CMN was estimated using a Differential scanning calorimeter (SIIO 6300 with auto-sampler, SIIO, Tokyo, Japan) equipped with an intercooler in order to assess the change in chemical properties of powders. Indium was used as a standard to calibrate the differential scanning calorimetry (DSC) temperature and enthalpy scale. The samples were hermetically sealed in aluminum pans and heated at a constant rate of 10 °C/min, over a temperature range of 0–350 °C. An inert atmosphere was maintained by purging with nitrogen at a flow rate of 100 mL/min.

##### Fourier Transform Infra-Red Spectroscopy (FTIR)

FTIR spectra were obtained after appropriate background subtraction using FTIR spectrophotometer (FTIR-8400SCE, Shimadzu Corporation, Tokyo, Japan) equipped with a diffuse reflectance accessory (DRS-8000, Shimadzu Corporation) and a data station. Previously dried powder samples of CMN and MSC CMN were mixed with dry potassium bromide and scanned within the range of 4000 to 400 cm^−1^.

##### *In Vitro* Cytotoxicity

Sulforhodamine B (SRB) assay utilized for performing *in vitro* cytotoxicity screening is based on the determination of cellular protein content of adherent as well as suspension cultures in 96-well format. One hundred milligrams of test sample (curcumin) was dissolved in 1 mL of DMSO and diluted up to 1 mg/mL. Further dilutions were made by using sterile deionized water to get 10, 20, 30, 40 µg/mL. The eight rows of 96-well culture plate were labeled as A, B, C, D, E, F, G, H. Ten microliters of the each test sample in 10% (*v*/*v*) DMSO in sterile deionized water was added to each compound well of a 96-well culture plate in rows B, C, D, E, respectively. In rows F and G (negative control well), 10 µL of 10% (*v*/*v*) DMSO was added. Ten microliters of standard doxorubicin in 10% (*v*/*v*) DMSO was added into each positive control well in row H. The cell monolayers were then removed from the medium and washed with sterilized phosphate buffer pH 7.4. The monolayer cell culture was trypsinized by adding 0.25% (*w*/*v*) trypsin in versene–EDTA (0.25 g EDTA and 1.5 mL of 0.5% (*w*/*v*) phenol red solution in 100 mL of phosphate buffer pH 7.4) and dispersed in 10 mL of culture medium. The cell concentration was adjusted to 1.9 × 10^4^ cells per well. One hundred and ninety microliters cell suspensions were added to the assay plates developed in previous step. Row A containing cell suspension was set aside for no-growth control (day 0). The plates were then incubated in a humidified incubator for 72 h at 37 °C with 5% CO_2_. The cell monolayers were fixed with 100 µL of cold 10% (*w*/*v*) tri chloroacetic acid by adding it to each well and incubated at 4°C for 1 h and dried. The cell monolayers were further stained with 100 µL of 0.057% (*w*/*v*) SRB solution. Unbound dye was removed by repeated washing using 1% (*v*/*v*) acetic acid, and then dried. Protein bound dye was extracted with 200 µL of 10 mM unbuffered Tris base (Tris hydroxyl methyl amino methane) solution from stained cells. The optical density was determined by using micro plate reader at 540 nm wavelength. The procedure was done in triplicate and repeated for MSC CMN. The percentage of cell growth inhibition was calculated by following equation.

(5) $$\text{\% Control of cell growth}=\frac{\text{mean OD samples}-\text{mean OD day }0}{\text{mean OD negative control}-\text{mean OD day }0}$$

where, OD in the optical density. A graph was plotted between percent control growth of cancer cells and concentration (µg/mL) of test samples [29].

#### 2.2.3. Preparation of Floating Tablets of MSC CMN

Tablets containing 50 mg of MSC CMN were prepared by direct compression method. The powder(s) of release retarding polymer (HPMC K 15M, sodium alginate) and gas forming agent (sodium bicarbonate) was passed through sieve #20, separately (Table 1). Mixing of powders was gently carried out using pestle and mortar for 10 min. MCC PH101 and magnesium stearate were then added to the powder mixture and mixing was continued for another 3 min. Finally, 400 mg of each mixture were weighed and fed manually into the die of a single punch tableting machine, equipped with flat-faced punches and tablets were prepared.

**Table 1:** Formulation design of gastroretentive floating tablets of MSC CMN (F1–F4) and CMN (CT).

Code	Drug(mg)	HPMC K 15M (mg)	Sodium Alginate (mg)	Sodium Bicarbonate (mg)	Citric Acid (mg)	MCC PH101 (mg)	Magnesium Stearate (mg)
F1	50	200	–	70	–	71	9
F2	50	–	200	70	–	71	9
F3	50	100	100	70	–	71	9
F4	50	100	100	70	5	66	9
CT	50	100	100	70	5	66	9

#### 2.2.4. Pharmacotechnical Characterization of Floating Tablets

The prepared floating tablets were evaluated for tablet thickness, hardness, friability test, weight variation, *in vitro* buoyancy, and *in vitro* release.

##### Thickness, Hardness, Friability and Weight Variation

A vernier calliper (least count = 0.01) was used to determine thickness of 10 randomly selected tablets. The result is expressed as mean values ± SD. The hardness of the tablets was determined using Monsanto hardness tester (HICON, Grover Enterprises, New Delhi, India), as expressed in kg/cm^2^. Three tablets were randomly picked from each formulation. Mean and standard deviation values were calculated. Friability of prepared tablets was determined by using Roche friabilator (HICON friability test apparatus). Ten tablets from each batch were selected randomly and weighed accurately. Tablets were then placed in a plastic chamber that rotates at 25 rpm, dropping tablets from a distance of six inches with each revolution. The friabilator was operated for 100 revolutions and the tablets were de-dusted and reweighed [30]. Friability was calculated using following equation:

% Friability = [(Initial weight − Final weight)/Initial weight] × 100 (6)

For weight variation test, twenty tablets of each batch were selected randomly and weighed. The average weight was calculated. The tablets passed the test if the individual weight of not more than 2 tablets deviated from the average weight by more than the percentage as per pharmacopoeial limits [30] and none deviated more than twice the percentage.

##### *In Vitro* Buoyancy

*In vitro* buoyancy studies were performed as per the method described by Rosa *et al.*, 1994 [31]. The randomly selected tablets (*n* = 3) from each formulation were placed in a 100 mL beaker containing phosphate buffer, pH 4.5. The time taken for the tablet to rise to the surface and float was taken as floating lag time.

##### *In Vitro* Drug Release

*In vitro* drug release study was conducted using Modified Rosette Rice apparatus [32]. The tablet was placed in modified beaker containing 70 mL of phosphate buffer, pH 4.5 with 0.02% *w*/*v* tween 80. A pH of 4.5 represents the midpoint of fed state pH ranging from 2.5 to 6.5 was selected. The test was performed at 37 ± 0.05 °C and 75 rpm. Five milliliters of sample was withdrawn at 0, 0.5, 1, 2, 3, 4, 6 and 8 h and filtered. An equal volume of fresh media was added into the beaker to maintain the sink condition. The samples were diluted appropriately and assayed spectrophotometrically at 426 nm to analyze the drug release in dissolution media. The study was done in triplicate. The data obtained was fitted to zero order, first order, Higuchi, Korsmeyer–Peppas and Hixson–Crowell release kinetic models to elucidate the release mechanism of drug from the tablet.

## 3. Results and Discussion

The utility of curcumin is limited by its low water solubility, and relatively low *in vivo* bioavailability. Based on its multiple therapeutic activities, intense research activities are in focus for developing an efficient delivery system of curcumin without these problems [33]. Particle engineering via melt sonocrystallization, which is a combination of melt solidification and ultra-sonication processes has advantages of melt solidified bonds and hard surface. This hardened surface enables the particles to withstand high sonication shear and maintain integrity even in highly porous form. Ultra sonication used in the technique is reported to cause spontaneous nucleation at relatively low degrees of super saturation, due to increase in the number of collisions. Further, ultrasonication enhanced collisions in molecules of the melt favors nucleation rather than crystallization. Thus, it may be concluded that MSC is a promising technique to obtain porous, amorphous material with high stability [34].

Practically speaking, it is a two-step process wherein the drug particles undergo melting and get immediately dispersed into fine particles with the help of ultrasonic energy [35]. In preparation of MSC CMN, the molten drug was sonicated for 4 min in a water bath sonicator. The time period is important, as longer sonication periods may lead to formation of aggregates that is undesirable because it may result in inappropriate physical properties of the compound [36]. The ultra-sonic wave frequency of 60 Kilohertz and a temperature of 70 to 80 °C were the major considerations of this technique that were optimized based on various preliminary studies (not reported). The melt sonocrystallized form of curcumin (MSC CMN) obtained as dry crystalline powder and stored in a desiccator protected from light. The percent yield was found to be 91.56%.

### 3.1. Evaluation

#### 3.1.1. Particle Size and Its Distribution

The mean particle size of the MSC CMN was reduced from 30.10 to 13.44 µm (CMN) as reported in Table 2. The quantum of reduction of the mean particle size was 55.35%.The explanation for reduction in particle size as found in literature is that application of ultrasonic energy to the melted form results in enhanced kinetic energy of molecules and further collision causes the drops to get reduced to micro-drops of narrow size range [37]. Correspondingly the specific surface area was found to increase by 35.34%; from 6937 (CMN) to 10730 cm^2^/mL (MSC CMN).

**Figure 1:**
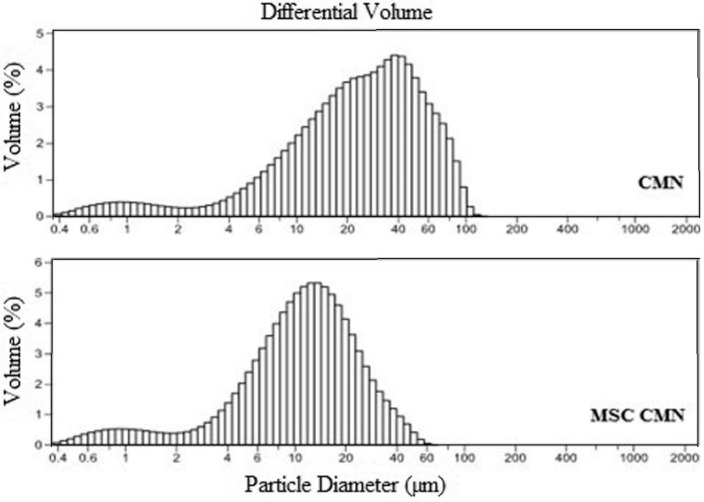
Frequency distribution curves of CMN and MSC CMN.

In particle size distribution analysis, skewness and kurtosis are the statistical parameters and are used for the analyses of micronized and controlled atomization as well as also for melt sonocrystallization [38]. The skewness of MSC CMN was reduced from 0.922 to 1.320 as compared to CMN (Figure 1) indicating that the particles of MSC CMN were more symmetrically distributed with respect to CMN. Similarly, the value of kurtosis for MSC CMN was found to increase from 0.290 to 2.154. Kurtosis specifies the shape of distribution and normal distribution of particles. Thus positive value of kurtosis in both the cases depicted peakedness of the distribution [39]. The modification in degree of peakedness as observed in case of MSC CMN was a result of sonocrystallization that normalized the distribution.

**Table 2:** Comparative micromeritic, rheological and solubility data of MSC CMN with reference to CMN.

Parameter		CMN	MSC CMN
Mean particle size (µm)		30.10	13.44
Standard deviation (µm)		22.57	9.646
Specific surface area (cm^2^/mL)		6937	10730
Skewness		0.922	1.320
Kurtosis		0.290	2.154
IQCS		0.203	0.166
Span		63.43	26.04
Variance (µm^2^)		509.4	93.04
Dynamic angle of repose (°)		38.21 ± 1.53	21.67 ± 1.72
Density (g/cc)	Bulk density	0.29 ± 1.48	0.49 ± 2.10
	Tapped density	0.43 ± 1.26	0.53 ± 1.81
Carr’s Index (%)		31.29 ± 1.62	18.49 ± 1.79
Hausner’s ratio		1.46 ± 1.13	1.23 ± 1.70
Solubility (µg/mL)	Distilled water	8.92 ± 1.41	21.14 ± 1.36
	Phosphate buffer, pH 4.5	6.301 ± 1.15	15.139 ± 1.23
Intrinsic dissolution rate (mg/cm^2^/min)	Distilled water	0.135 ± 1.39	0.242 ± 1.42
	Phosphate buffer, pH 4.5	0.098 ± 1.50	0.195 ± 1.26

Other parameters derived from particle distribution data are span and polydispersity index. A high span value indicates a wide size distribution with large particles and a high polydispersity index [40]. Conventional particle size reduction methodology may result in large span value but the melt sonocrystallization technique provides an opportunity for producing powder of low span value that indicates narrow particle size distribution and uniformity [39]. The values of variance in case of original and MSC form were observed to be 509.4 and 93.04 µm^2^, respectively. Lower variance of MSC CMN specified less variability or lies near to the mean in comparison to CMN particle sized distribution.

#### 3.1.2. Flow Properties

Improvement in flowability and packing properties of powder particles is essential and can be assessed by angle of repose [27]. Angle of repose in case of CMN was 38.21° ± 1.53°, which specified that it possessed very poor flow property, whereas for MSC CMN, it was found to be 21.67° ± 1.72° (Table 2). Thus, MSC technique results in improvement of flow properties [25]. The packing characteristics defined by bulk density and tapped density were higher for of MSC CMN than the original form of CMN suggesting lesser package volume for MSC CMN than CMN. The important factors that affect bulk density of powder and its flow properties are inter-particulate interaction including friction and adhesion. Melt sonocrystallized form of drug showed reduction in inter-particulate interaction due to decrease in surface roughness, thus better flow properties [25]. The compressibility indicators: Carr’s compressibility index and Hausner’s ratio of MSC CMN were lower than their CMN.

#### 3.1.3. Equilibrium Solubility

The equilibrium solubility study was performed in distilled water and phosphate buffer, pH 4.5. The selection of latter media was based on studying the effect of melt sonocrystallization on the solubility of curcumin at the pH of proposed site of drug action. The study revealed 2.36-fold and 2.40-fold enhancement in solubility in distilled water and phosphate buffer, pH 4.5, respectively (Table 2).While the magnitude of solubility enhancement was same irrespective of the media, the basal values of solubility were slightly different while the enhanced values were significantly (*p* < 0.05) different. The solubility enhancement was attributed to the fact that reduction in particle size affected the specific surface area and hence the solubility [41]. Another explanation may be that the application of ultrasonic energy may have caused surface of the particles to fracture (as shown in SEM photomicrograph late), thus, allowing increased solvent uptake [42].

#### 3.1.4. Intrinsic Dissolution Rate

**Figure 2:**
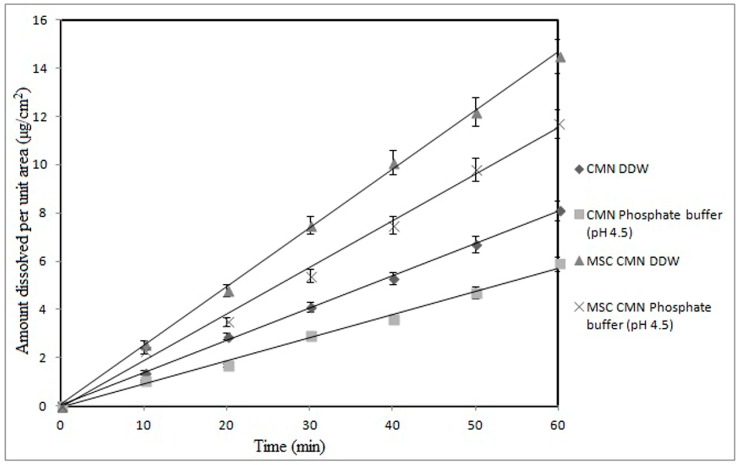
Intrinsic dissolution rate profile of CMN and MSC CMN in different dissolution media.

Measurement of intrinsic dissolution rate (IDR) is an important tool in pharmaceutical research allowing characterization of the different crystal forms of drugs by exposing a constant surface area to the dissolution medium. It is useful in predicting absorption problems due to dissolution rate [43]. The IDR of CMN was 1.35 ± 1.39 mg/cm^2^/min in distilled water and 0.098 ± 1.50 mg/cm^2^/min in phosphate buffer, pH 4.5, which increased to 0.242 ± 1.42 and 0.195 ± 1.26 mg/cm^2^/min, respectively, for MSC CMN. The IDR profiles of CMN and MSC CMN (Figure 2) evidence the effect of physical form and pH on the IDR. Clearly, The MSC CMN displayed higher IDR in comparison to CMN in both the media. Furthermore the IDR in phosphate buffer, pH 4.5 was lower than distilled water (pH approximately 6.8) for both forms. This is indicative of higher dissolution in intestine than gastric cavity; however, absorption problems are not predicted. Higher IDR of MSC CMN from a disc of constant surface area is attributable to porous nature of the MSC CMN crystals that facilitated ingress of dissolution medium in the disc matrix leading to higher IDR [44].

From this result it could be expected that MSC CMN will exhibit comparatively better dissolution rate and consequently, better absorption than CMN.

#### 3.1.5. X-ray Diffraction (XRD)

**Figure 3:**
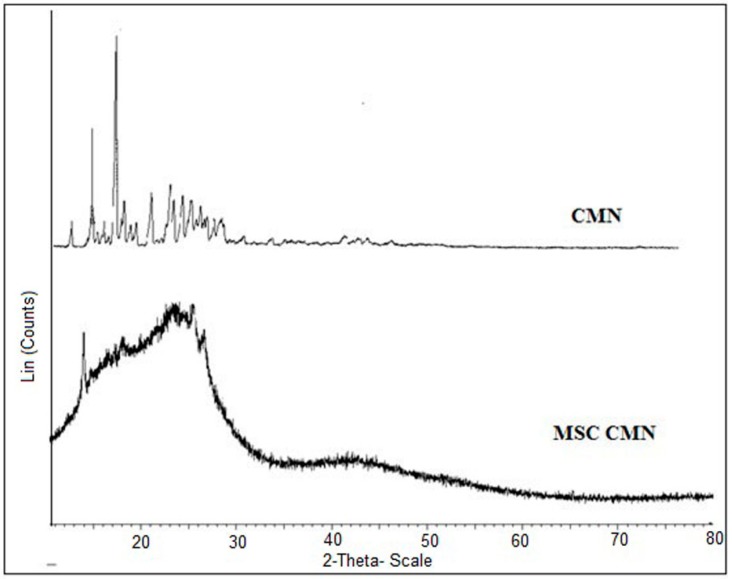
XRD spectra of CMN and MSC CMN.

XRD is the technique employed for the identification of crystalline solid phases. On comparing the diffractogram of CMN with that of MSC CMN, the diffraction peaks of CMN were clearly detectable in MSC CMN (Figure 3), suggesting that the MSC CMN did not undergo any modification in the crystal habit and this also confirmed the absence of new reflections and other crystal phases. Notably, differences in the relative intensity of peaks were detectable. The intensity of peaks of MSC CMN was lesser than CMN. This may be due to differences in the crystallinity or particle size of the samples. Therefore, the relative abundance of the planes exposed to X-ray source had altered, producing variations in relative intensities of the peak.

#### 3.1.6. Scanning Electron Microscopy

**Figure 4:**
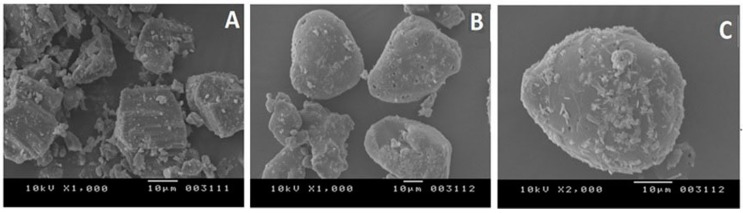
Scanning Electron Micrograph of (**A**) CMN at 1000×; (**B**) MSC CMN at 1000×; and (**C**) MSC CMN at 2000×.

Scanning electron microscopy was used to visualize the morphology of original as well as melt sonocrystallized forms of drug. SEM images of CMN (Figure 4A) showed the presence of large prismatic-cube crystalline particles with rough surface edges. Slight imperfections on planar surface were impressions/remnants of the conventional crystallization process [45]. The MSC CMN (Figure 4B,C) particles were observed to be regular, uniform and spheroidal in shape with pores in their crystalline structure. Some pitted and shrunken areas were also visualized on the surface of MSC CMN particles as sonication of the molten mass may create cracks, reduce the particle size and increase the intra-particulate porosity. Similar results have been reported by El Kamel *et al.* [44] for flurbiprofen and Maheshwari *et al.* [41] for ibuprofen. Thus, sonocrystallization can be considered as an effective tool to affect surface morphology and structure of crystalline form of drug [46].

#### 3.1.7. Differential Scanning Calorimetry (DSC)

**Figure 5:**
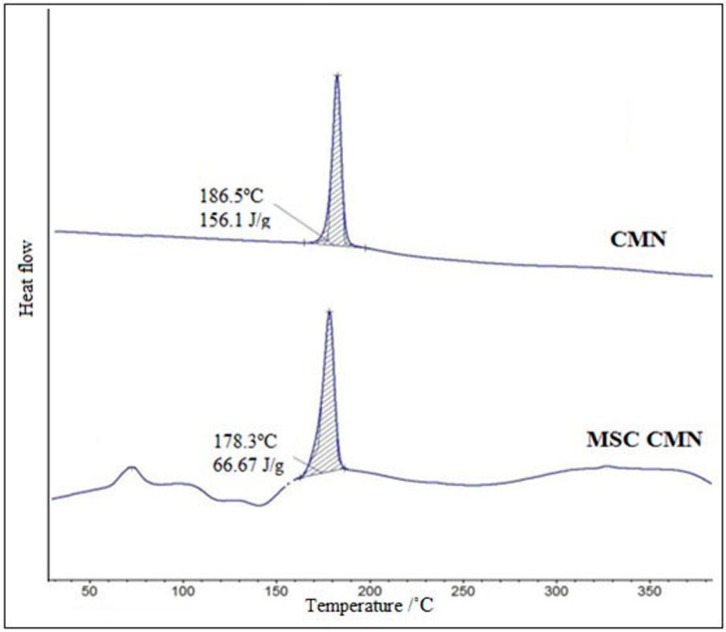
DSC thermogram profile of CMN and MSC CMN.

The DSC scan of CMN exhibited a single endotherm at 186.5 °C with a normalized energy of 156.1 J/g (Figure 5). The thermogram of MSC CMN showed a characteristic crystallization peak at 178.3°C, with a normalized energy of 66.67 J/g. The sharp endotherm with the minor shift in melting point was obtained, which may be caused due to reduction in crystal size. In the report by Maheshwari *et al.*, [41], where changes in thermal properties of MSC ibuprofen were documented as broadening and asymmetry of endothermic peak, the same is not true in our experiment. Neither broadening, nor asymmetry of the peak was observed, but considerable reduction in the ∆*H* values of MSC CMN was indicative of small porous crystals. The crystallization peak at 178.3 °C in thermogram of MSC CMN revealed the formation of polymorph form 2, which was in accordance with the study reported by Sanphui *et al.* [47].

#### 3.1.8. Fourier Transformed Infra-Red Spectroscopy (FTIR)

**Figure 6:**
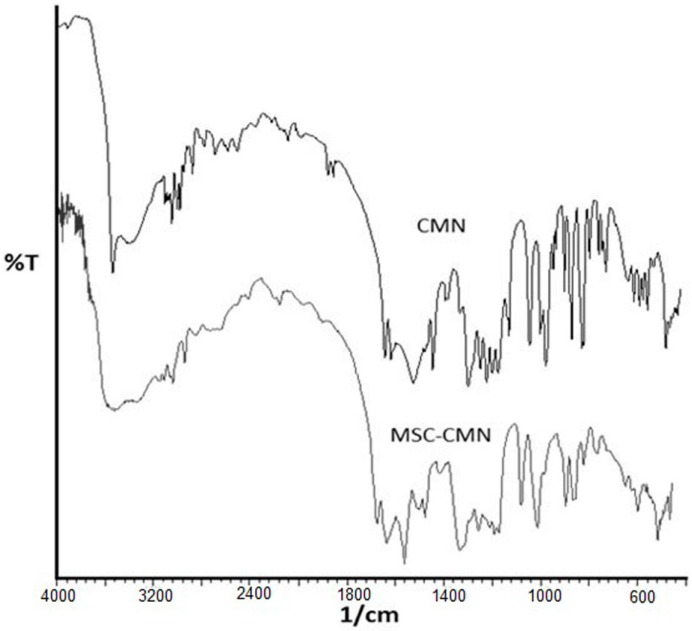
FTIR spectra of CMN and MSC CMN.

The FTIR spectrum of CMN showed the presence of vibrations at 3504.66 cm^−1^ due to O–H stretching [48]. The peak at 1627.92 cm^−1^ was observed due to stretching vibrations of C=O bond. The presence of peak at 1602.04 cm^−1^ is due to C=C functions and at 1230.44 cm^−1^ is due to C–O–C (Figure 6). The FTIR spectrum of MSC CMN was almost an overlapping spectrum of CMN. Thus, indicating that no transformation in chemical structure of drug occurred due to the processing steps [21].

#### 3.1.9. *In Vitro* Cytotoxicity

**Figure 7:**
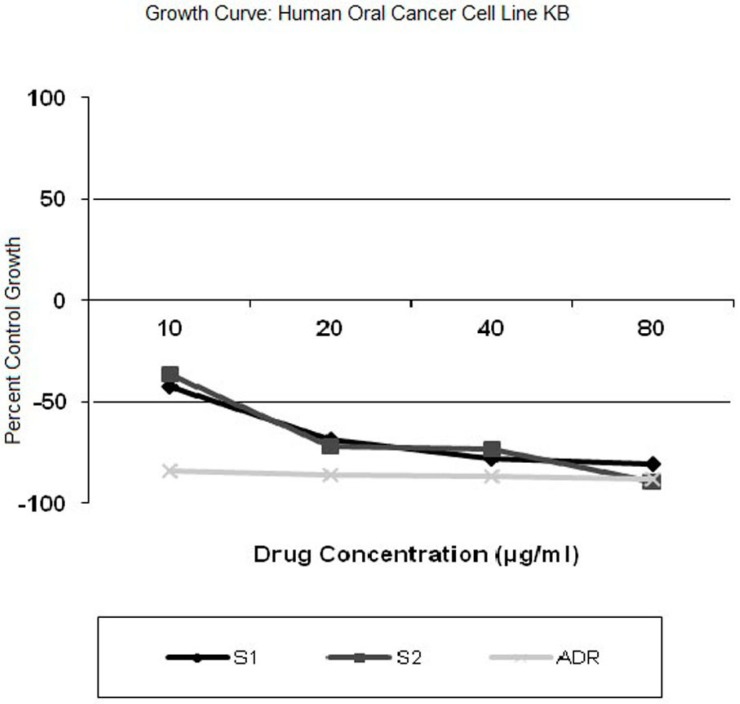
*In vitro* cytotoxicity profile of curcumin (S1), MSC Curcumin (S2), doxorubicin (ADR) in human cancer cell line KB.

In *in vitro* cytotoxic studies, doxorubicin was used as reference standard against pure and MSC form of curcumin. The results (Figure 7) exhibited the sensitivity of cancer cell line to enhancing serial concentration of curcumin, MSC curcumin and doxorubicin, followed by calculation of percentage growth inhibition with respect to GI_50_ values for all the treatment groups. The percent control growth of cancer cells reduced from −42.2% to −80.4% and −84.0% and −88.2% as the concentration increased from 10 to 80 µg/mL for curcumin and doxorubicin, respectively. The percent control growth of cancer cells decreased from −36.6% to −89.6% in same concentrations. It is a known fact that GI_50_ of less than 10 indicates greater cytotoxic activity for pure compounds, whereas GI_50_ of less than 20 indicates cytotoxic activity for compounds other than pure ones [29]. GI_50_ value of doxorubicin, CMN and MSC CMN was found to be less than 10, specifying inhibition of cancerous cell growth more effectively at its least concentration by 50%. This may be attributed due to the fact that CMN ensured higher uptake of the drug by the cancerous cells. Similar value of GI_50_ in case of MSC CMN reveals that the therapeutic activity of curcumin is retained in its MSC form, exhibiting same cytotoxic effect.

LC_50_ (50% lethal concentration) of CMN and MSC CMN was found to be 41.4 and 40.4, respectively, whereas doxorubicin exhibited the LC_50_ value of 31.7. TGI (total growth inhibition) value was depicted to be 9.5 for CMN and 10.8 for MSC CMN quite similar to that of reference drug doxorubicin possessing TGI value less than 10. Thus, specifying the optimum anticancer activity with respect to the reference drug taken.

### 3.2. Product Development

#### 3.2.1. Gastroretentive Floating Tablets of MSC CMN

Gastroretentive floating tablets of MSC CMN were developed as these systems would have the advantage of delivering curcumin in the gastric cavity, where improved residence time could prove advantageous for exerting local action for treatment of cancer. Compared to conventional dosage forms, gastro retentive drug delivery systems (GRDDS) are designed to remain in the stomach for a prolonged, predictable time. Consequently, gastric residence time of drug substances is extended and bioavailability improved. Floating tablets have also been reported by Abbaraju *et al.* employing mesoporous silica nanoparticles in order to improvise the drug delivery of both hydrophobic and hydrophilic drugs in comparison to conventional floating tablets [49]. Thus, the aim behind developing floating tablets of MSC CMN was two pronged: (i) where MSC technology increased the solubility of curcumin, thus enhancing dissolution and (ii) gastroretentive formulation would localize the improved form of drug at the site of action. Thus, this system would have a combinatorial effect of both melt sonocrystallization and gastro retentive floating system in enhancing the oral bioavailability of curcumin. Floating tablets were formulated by using release-retarding polymer HPMC K 15M and sodium alginate, and gas forming agent (sodium bicarbonate). In all four formulations of gastroretentive floating tablets of MSC CMN (F1–F4) were formulated and evaluated for physical and floating features. The optimized MSC CMN floating tablet was further characterized for *in vitro* release and compared with control tablet (CT).

#### 3.2.2. Pharmacotechnical Characterization of Floating Tablets

##### Thickness, Hardness, Friability and Weight Variation

The thickness of the tablets ranged between 2.98 ± 1.19 and 3.02 ± 1.13 mm and the diameter was between 8.05 ± 1.20 and 8.05 ± 1.61 mm (Table 3) suggesting easy handling of the tablets. The tablets passed the weight variation test as specified in IP [30]. It was observed that not more than two tablets deviated from the average weight by more than 5% and none deviated more than twice of the average weight. The hardness of 3.50 ± 1.52 kg/cm^2^ and friability less than 1%, assured sufficiently strong tablets to withstand the distribution and storage vibrations.

**Table 3:** Physicochemical properties of the MSC CMN gastro-retentive floating tablets.

Code	Thickness (mm)	Weight (mg)	Diameter (mm)	Hardness (kg/cm^2^)	Friability (%)	Floating Lag Time (min)	Floating Duration (h)
F1	3.01 ± 1.24	398 ± 1.16	8.050 ± 1.20	3.37 ± 1.23	0.56 ± 1.64	120 ± 1.38	7.50 ± 1.12
F2	2.98 ± 1.19	397 ± 1.58	8.052 ± 1.17	3.33 ± 1.53	0.53 ± 1.39	60 ± 1.53	9.45 ± 1.15
F3	3.00 ± 1.27	398 ± 1.16	8.054 ± 1.61	3.58 ± 1.78	0.59 ± 1.15	3.0 ± 1.09	11.30 ± 1.21
F4	3.02 ± 1.13	399 ± 1.85	8.051 ± 1.14	3.50 ± 1.52	0.57 ± 1.36	Within 3 s	18.26 ± 1.35

##### *In Vitro* Buoyancy

The formulations F1–F3 showed a lag time of about 3.0 ± 1.09 to 120 ± 1.38 min whereas formulation F4 displayed least floating lag time of 3 s, which can be attributed to the presence of citric acid, a gas generating agent. The generated gas was trapped and protected within the gel matrix formed by hydration of the polymer (s), thus reducing the density of the tablet. Tablets become buoyant when their density falls to less than 1 [50]. Formulation F1 showed maximum lag time as the release medium penetrates into the tablet followed by the swelling and gel formation of hydrophilic polymeric chains of HPMC K 15M. Thus, higher value of lag time (120 ± 1.38 min) was due to the time required for appropriate expansion of tablets. The CO_2_ bubbles and swollen particles of swelling agents were entrapped inside gel matrix of hydrophilic polymer and as a result the density reduces providing buoyancy to tablets. Further, reduction in concentration of polymer resulted in lower lag time values as in case of F3 (3.0 ±1.09 min) and F4 formulation [51]. Thus, formulation F4 was selected as the optimized formulation because it afforded best *in vitro* buoyancy and was subjected to *in vitro* release study.

##### *In Vitro* Drug Release

**Figure 8:**
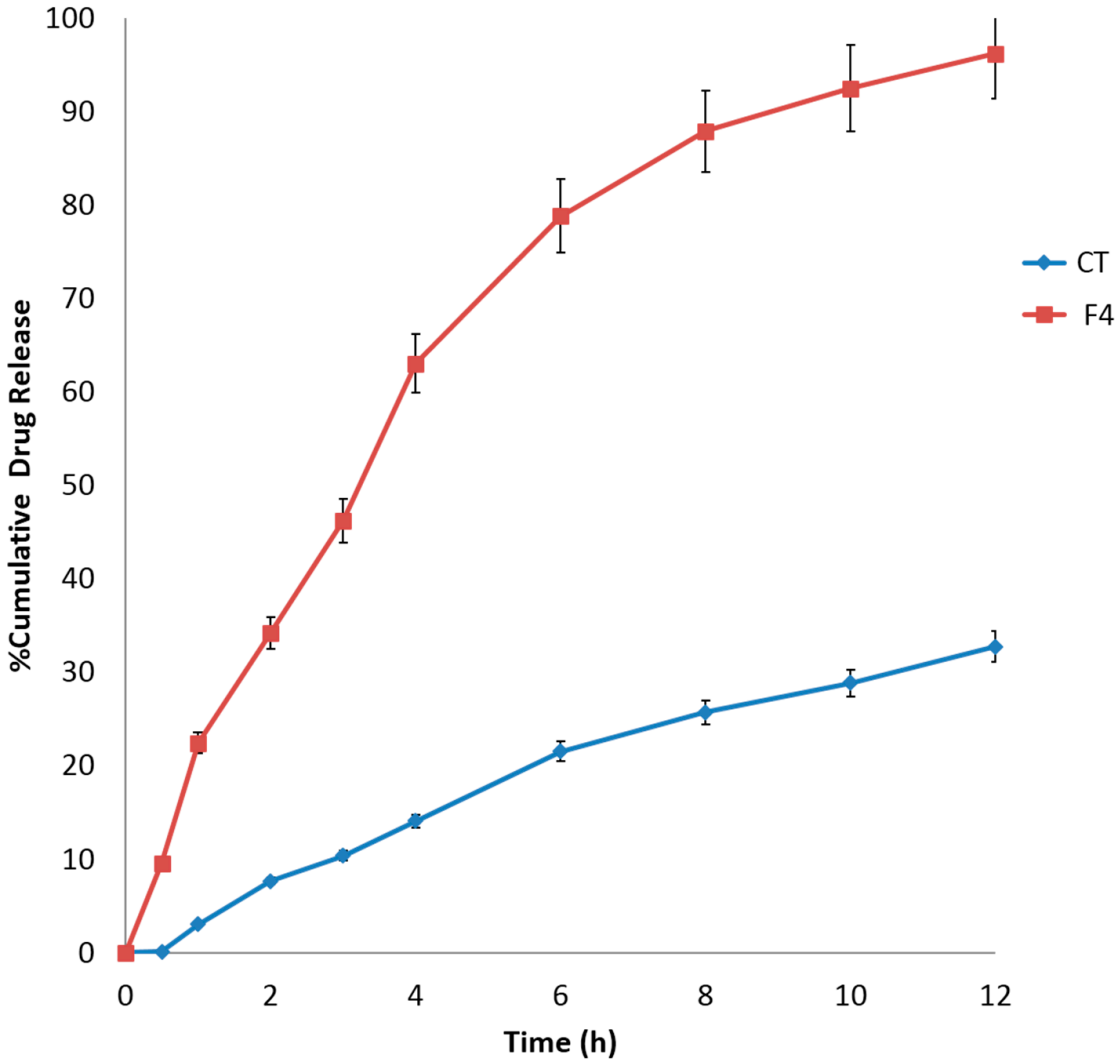
Percent Cumulative drug release profile of Gastro-retentive floating tablet of curcumin (CT) and F4 formulation in phosphate buffer, pH 4.5.

The *in vitro* drug release of MSC CMN from F4 was compared against CMN tablet (CT) that was of same composition with respect to the excipients. The cumulative drug release from CT was 32.74% ± 1.57%, whereas F4 displayed a cumulative drug release of 96.22% ± 1.43% in 12 h (Figure 8). The drug release from F4 gradually increased with almost complete sustained release for 12 h, whereas CT showed slow incomplete release in the same time period. In terms of composition both the tablets were the same, except for the physical form of curcumin. In F4, the melt sonocrystallized form of the drug afforded higher solubility and hence higher dissolution. On the other hand, conventional curcumin crystals presented solubility-limited dissolution. The *in vitro* release data was fitted to various kinetic models: zero order, first order, Higuchi, Korsmeyer–Peppas and Hixson–Crowell release kinetic model. The coefficient of determination (*r*^2^) was found to be maximum for Higuchi model (*r*^2^, 0.9663), which specifies that diffusion as the mechanism for drug release [52] from F4 floating tablets. For CT tablets, the data best fitted Korsmeyer–Peppas model with *r*^2^ value of 0.9826 and “*n*” value less 1 (0.9429).

On comparing the *in vitro* release profiles with that of control tablet (CT) using one-way ANOVA significant difference (*p* < 0.05) in drug release was observed. The similarity factor (*f2*) and dissimilarity factor (*f1*) were also calculated. With reference to control tablet (CT), MSC CMN tablet (F4) showed high dissimilarity (*f2* = 15) between two profiles. The *f2* value of less than 50 implied dissimilarity between the release profiles.

## 4. Conclusions

The present study revealed that the application of melt sonocrystallization to improve the flow properties, enhance the solubility and dissolution of poorly water-soluble drug curcumin. The *in vitro* cytotoxic potential of curcumin was retained in its sonocrystallized form and was comparable to doxorubicin. The melt sonocrystallized form when developed as a gastroretentive floating tablet exhibited appropriate *in vitro buoyancy* with superior dissolution characteristics than conventional form of curcumin. Thus, it was concluded that product development of melt sonocrystallized curcumin could prove to be a superior system for the treatment of gastric cancer.
